# Miscellany

**Published:** 1909-12

**Authors:** 


### MISCELLANY.

---

Army Medical Corps Examinations at Washington, Chicago and San Francisco.—The Surgeon General of the Army announces that the War Department has appointed permanent boards for the preliminary examination of applicants for appointment in the medical corps of the army, to meet at Washington, D. C., Fort Sheridan (near Chicago), Illinois and San Francisco, California, in addition to the usual preliminary examination boards that are assembled at various army posts throughout the United States from time to time. The permanent boards will hold sessions on the second Monday of each month.

A limited number of successful candidates will be appointed first lieutenants in the Medical Reserve Corps (salary, $2,000 per annum), and assigned to army posts until the next session of the Army Medical School, when they will be ordered to attend the school as "student candidates." Applicants must be citizens of the United States, between twenty-two and thirty years of age, graduates of reputable medical schools, of good moral character and habits, and shall have had a year's hospital training after graduation, or its equivalent.

Full information concerning the examination can be procured upon application to the "Surgeon General, U. S. Army, Washington, D. C.

---

Army Medical Corps Examinations.—The surgeon general of the army announces that the first of the preliminary examinations for the appointment of first lieutenants in the Army Medical Corps for the year 1910 will be held on January 17, 1910, at points to be hereafter designated.

Full information concerning the examination can be procured upon application to the Surgeon General, United States Army, Washington, D. C. The essential requirements to securing an invitation are that the applicant shall be a citizen of the United States, shall be between twenty-two and thirty years of age, a graduate of a medical school legally authorized to confer the degree of doctor of medicine, shall be of good moral character and habits, and shall have had at least one year's hospital training or its equivalent in practice after graduation. The examinations will he beld concurrently throughout the country at points where boards can be convened. Due consideration will be given to localities from which applications are received, in order to lessen the traveling expenses of applicants as much as possible.

The examination in subjects of general education (mathematics, geography, history, general literature, and Latin), may be omitted in the case of applicants holding diplomas from reputable literary or scientific colleges, normal schools or high schools, or graduates of medical schools which require an entrance examination satisfactory to the faculty of the Army Medical School.

In order to perfect all necessary arrangements for the examination, applications must be complete and in possession of the Adjutant General on or before January 3, 1910. Early attention is therefore enjoined upon all intending applicants. There are at present eighty-one vacancies in the Medical Corps of the Army.

